# *rac*-(3*S*,4*S*)-3-Hy­droxy-4-phenyl-1-[(*S*)-(3-phenyl-4,5-dihydro-1,2-oxazol-5-yl)meth­yl]-4,5-dihydro-1*H*-1,5-benzo­diazepin-2(3*H*)-one

**DOI:** 10.1107/S1600536811010129

**Published:** 2011-03-23

**Authors:** Mohamed Rida, El Mokhtar Essassi, Stéphane Massip, Saïd Lazar, Hafid Zouihri

**Affiliations:** a Laboratoire de Chimie Hétérocyclique, Pole de Compétence PHARCHIM, Université Mohammed V-Agdal, BP 1014 Rabat, Morocco; b Institut of Nanomaterials and Nanotechnology (INANOTECH), Avenue de l Armée Royale, Rabat, Morocco; c Laboratoire de Chimie Physique et Minérale, Service de Cristallographie, Université Victor Ségalen Bordeaux 2, Bordeaux Cedex, France; d Laboratoire de Biochimie, Environnement et Agroalimentaire (URAC 36), Faculté des Sciences et Techniques Mohammedia, Université Hassan II Mohammedia-Casablana, BP 146, 20800 Mohammedia, Morocco; e Laboratoires Diffraction des Rayons X sur Monocristal, Centre Nationale pour la Recherche Scientifique et Technique, Rabat, Morocco

## Abstract

In the title compound, C_25_H_23_N_3_O_3_, the seven-membered diazepine ring adopts a boat conformation with the hydroxy-substituted C atom at the prow and fused-ring C atoms at the stern. The crystal packing features C—H⋯O, C—H⋯π and N—H ⋯π inter­actions

## Related literature

For the preparation and biological activity of benzodiazepines, see: Ahabchane *et al.* (1999 ▶), Grunewald *et al.* (1996 ▶); Ding *et al.*, (1999 ▶). For related structures, see: Saber *et al.* (2010*a* ▶,*b* ▶); Ballo *et al.* (2010 ▶). For puckering parameters, see: Cremer & Pople (1975 ▶).
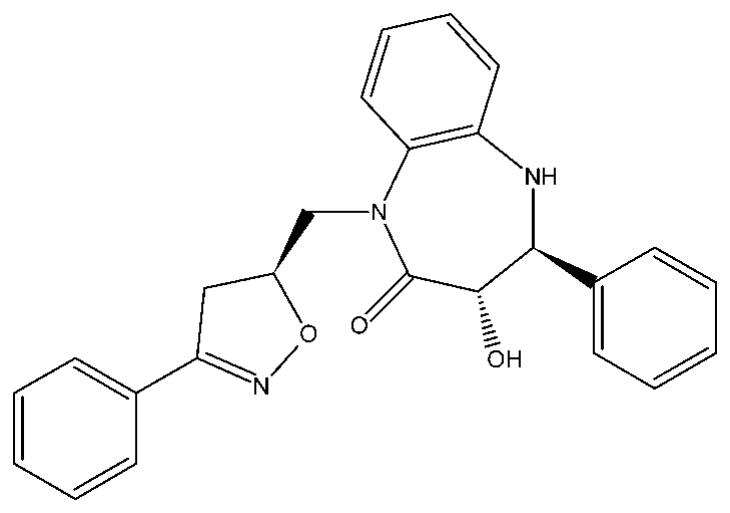


## Experimental

### 

#### Crystal data

C_25_H_23_N_3_O_3_
*M* *_r_* = 413.46
Triclinic, 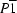

*a* = 8.981 (2) Å
*b* = 9.044 (2) Å
*c* = 13.685 (1) Å
α = 95.373 (10)°
β = 102.433 (10)°
γ = 100.03 (2)°
*V* = 1058.9 (3) Å^3^
*Z* = 2
Cu *K*α radiation
μ = 0.70 mm^−1^
*T* = 293 K
0.15 × 0.10 × 0.08 mm

#### Data collection

Enraf–Nonius CAD-4 diffractometer
Absorption correction: ψ scan (North *et al.*, 1968 ▶) *T*_min_ = 0.903, *T*_max_ = 0.946
3584 measured reflections
3584 independent reflections
3232 reflections with *I* > 2σ(*I*)
*R*_int_ = 0.000
2 standard reflections every 90 min  intensity decay: none

#### Refinement

*R*[*F* ^2^ > 2σ(*F* ^2^)] = 0.050
*wR*(*F* ^2^) = 0.157
*S* = 1.05
3584 reflections
286 parameters
1 restraint
H atoms treated by a mixture of independent and constrained refinement
Δρ_max_ = 0.20 e Å^−3^
Δρ_min_ = −0.17 e Å^−3^

### 

Data collection: *CAD-4 Software* (Enraf–Nonius, 1989 ▶); cell refinement: *CAD-4 Software*; data reduction: *CAD-4 Software*; program(s) used to solve structure: *SHELXS97* (Sheldrick, 2008 ▶); program(s) used to refine structure: *SHELXL97* (Sheldrick, 2008 ▶); molecular graphics: *PLATON* (Spek, 2009 ▶); software used to prepare material for publication: *publCIF* (Westrip, 2010 ▶).

## Supplementary Material

Crystal structure: contains datablocks I, global. DOI: 10.1107/S1600536811010129/ld2003sup1.cif

Structure factors: contains datablocks I. DOI: 10.1107/S1600536811010129/ld2003Isup2.hkl

Additional supplementary materials:  crystallographic information; 3D view; checkCIF report

## Figures and Tables

**Table 1:** Hydrogen-bond geometry (Å, °) *Cg*4 is the centroid of the C26–C31 ring.

*D*—H⋯*A*	*D*—H	H⋯*A*	*D*⋯*A*	*D*—H⋯*A*
C16—H16⋯O22	0.93	2.44	3.206 (3)	140
C25—H25*a*⋯O14^i^	0.97	2.59	3.493 (3)	154
C27—H27⋯O15^ii^	0.93	2.40	3.302 (3)	162
C28—H28⋯O22^i^	0.93	2.52	3.152 (2)	125
C19—H19⋯*Cg*4^iii^	0.93	2.78	3.534 (3)	139
N13—H13⋯*Cg*4^iii^	0.93 (2)	2.77 (2)	3.671 (3)	164

Symmetry codes: (i) 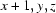; (ii) 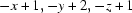; (iii) 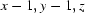.

**Fig. 1.**
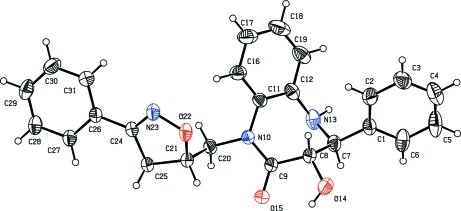
Molecular view of the title compound showing the atom-labeling scheme. Displacement ellipsoids are drawn at the 50% probability level. H atoms are represented as small spheres of an arbitrary radius.

**Fig. 2.**
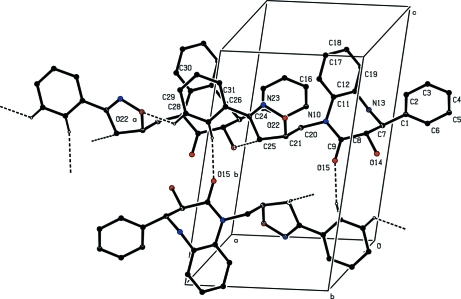
Partial packing view showing chains formed by C—H···O hydrogen bondings. H atoms not involved in the hydrogen bonds have been omitted for clarity.

## supplementary crystallographic information

### Comment

Benzodiazipines have found wide-spread clinical use as inhibitors of phenylethanolamine N-methyltransferase [Grunewald *et al.*, 1996] and inhibitors of farnesyltransferase [Ding *et al.*, 1999]. In view of their importance and to ascertain molecular conformation [Ahabchane *et al.*, 1999. Saber *et al.* (2010*a* and 2010*b*) and Ballo *et al.* (2010)], a crystallographic study of the title compound has been carried out.

The molecular structure of the title compound is depicted in Fig.1. The seven-membred ring adopts a boat conformation with equatorial orientations of both phenyl and hydroxyl substituents. Its puckering parameters [Cremer & Pople, 1975] are q_2_ = 0.997 (2) Å, q_3_ = 0.1541 (19) Å, φ_2_ = 317.05 (11)°, φ_3_ = 185.2 (8)°.

Phenyl substituent at C24 atom is rotated out of the plane of the 4,5-dihydro-1,2-oxazole ring by 20.1 (1)°.

The crystal packing is controlled by C—H···O H-bonds and C—H ···π and N—H ···π interactions involving the C26-C31 (Cg4) benzene ring (Table 1).

### Experimental

A solution of NaOCl (10 ml) was added to a mixture of 1-allylmethyl-3-hydroxy-4-phenyltetrahydro-1,5-benzodiazepin-2-one (2.58 g, 0.01 mmol) and benzaldoxime (1.21 g, 0.01 mmol) in chloroform (30 ml) under vigorous stirring. After 3 h, the organic layer was washed with water and dried by addition of anhydrous MgSO_4_. After removal of the solvent by evaporation, the crude residual mixture obtained was purified by column chromatography on a silica gel column and eluted with an eluent composed of ether/petroleum ether (1/9). Crystals was obtained when solvent was allowed to evaporate.

### Refinement

All O–H and C—H H-atoms were placed in calculated positions (C—H: 0.93 to 0.98 Å and O–H = 0.82) and allowed to ride on their parent atoms, with U (H) set to 1.2 U(C) and 1.5 U(O). Torsion angle for the hydroxyl group was optimized from electron density.

The amino H-atom was located in a difference Fourier map and was refined independently.

### Crystal data

**Table:**

C_25_H_23_N_3_O_3_	*Z* = 2
*M**_r_* = 413.46	*F*(000) = 436
Triclinic, *P*1	*D*_x_ = 1.297 Mg m^−^^3^
Hall symbol: -P 1	Cu *K*α radiation, λ = 1.54180 Å
*a* = 8.981 (2) Å	Cell parameters from 25 reflections
*b* = 9.044 (2) Å	θ = 25–35°
*c* = 13.685 (1) Å	µ = 0.70 mm^−^^1^
α = 95.373 (10)°	*T* = 293 K
β = 102.433 (10)°	Prism, colourless
γ = 100.03 (2)°	0.15 × 0.10 × 0.08 mm
*V* = 1058.9 (3) Å^3^	

### Data collection

**Table:**

Enraf–Nonius CAD-4 diffractometer	3232 reflections with *I* > 2σ(*I*)
Radiation source: fine-focus sealed tube	*R*_int_ = 0.0000
graphite	θ_max_ = 64.9°, θ_min_ = 5.0°
ω–2θ scans	*h* = −10→10
Absorption correction: ψ scan (North *et al.*, 1968)	*k* = −10→10
*T*_min_ = 0.903, *T*_max_ = 0.946	*l* = 0→16
3584 measured reflections	2 standard reflections every 90 min
3584 independent reflections	intensity decay: none

### Refinement

**Table:**

Refinement on *F*^2^	Secondary atom site location: difference Fourier map
Least-squares matrix: full	Hydrogen site location: inferred from neighbouring sites
*R*[*F*^2^ > 2σ(*F*^2^)] = 0.050	H atoms treated by a mixture of independent and constrained refinement
*wR*(*F*^2^) = 0.157	*w* = 1/[σ^2^(*F*_o_^2^) + (0.0902*P*)^2^ + 0.363*P*] where *P* = (*F*_o_^2^ + 2*F*_c_^2^)/3
*S* = 1.05	(Δ/σ)_max_ = 0.005
3584 reflections	Δρ_max_ = 0.20 e Å^−^^3^
286 parameters	Δρ_min_ = −0.17 e Å^−^^3^
1 restraint	Extinction correction: *SHELXL97* (Sheldrick, 2008), Fc^*^=kFc[1+0.001xFc^2^λ^3^/sin(2θ)]^-1/4^
Primary atom site location: structure-invariant direct methods	Extinction coefficient: 0.086 (5)

### Special details

**Table:**

Geometry. All e.s.d.'s (except the e.s.d. in the dihedral angle between two l.s. planes) are estimated using the full covariance matrix. The cell e.s.d.'s are taken into account individually in the estimation of e.s.d.'s in distances, angles and torsion angles; correlations between e.s.d.'s in cell parameters are only used when they are defined by crystal symmetry. An approximate (isotropic) treatment of cell e.s.d.'s is used for estimating e.s.d.'s involving l.s. planes.
Refinement. Refinement of *F*^2^ against ALL reflections. The weighted *R*-factor *wR* and goodness of fit *S* are based on *F*^2^, conventional *R*-factors *R* are based on *F*, with *F* set to zero for negative *F*^2^. The threshold expression of *F*^2^ > σ(*F*^2^) is used only for calculating *R*-factors(gt) *etc*. and is not relevant to the choice of reflections for refinement. *R*-factors based on *F*^2^ are statistically about twice as large as those based on *F*, and *R*- factors based on ALL data will be even larger.

### Fractional atomic coordinates and isotropic or equivalent isotropic displacement parameters (Å^2^)

**Table:**

	*x*	*y*	*z*	*U*_iso_*/*U*_eq_	
O14	−0.14283 (18)	0.8107 (2)	0.54940 (13)	0.0717 (5)	
O15	0.1367 (2)	0.8258 (3)	0.51556 (12)	0.0931 (7)	
O22	0.45964 (14)	1.08367 (16)	0.74426 (11)	0.0562 (4)	
N10	0.25123 (17)	0.78999 (18)	0.67247 (11)	0.0479 (4)	
N13	0.0378 (2)	0.52139 (19)	0.67025 (15)	0.0621 (5)	
N23	0.61410 (18)	1.11628 (19)	0.80393 (13)	0.0534 (4)	
C1	−0.2338 (2)	0.5566 (2)	0.66492 (17)	0.0546 (5)	
C2	−0.2327 (3)	0.6165 (3)	0.76241 (19)	0.0742 (7)	
C3	−0.3594 (3)	0.5798 (4)	0.8033 (2)	0.0860 (8)	
C4	−0.4916 (3)	0.4842 (3)	0.7485 (3)	0.0888 (9)	
C5	−0.4981 (3)	0.4269 (3)	0.6516 (3)	0.0944 (10)	
C6	−0.3691 (3)	0.4630 (3)	0.6095 (2)	0.0768 (7)	
C7	−0.0924 (2)	0.5884 (2)	0.62070 (16)	0.0550 (5)	
C8	−0.0343 (2)	0.7579 (2)	0.62179 (15)	0.0521 (5)	
C9	0.1256 (2)	0.7912 (2)	0.59864 (14)	0.0560 (5)	
C11	0.2384 (2)	0.7330 (2)	0.76507 (14)	0.0478 (5)	
C12	0.1330 (2)	0.5989 (2)	0.76299 (17)	0.0543 (5)	
C16	0.3373 (3)	0.8063 (3)	0.85519 (15)	0.0599 (5)	
C17	0.3345 (3)	0.7461 (4)	0.94383 (18)	0.0787 (8)	
C18	0.2311 (4)	0.6129 (4)	0.9423 (2)	0.0884 (9)	
C19	0.1311 (3)	0.5418 (3)	0.8540 (2)	0.0733 (7)	
C20	0.4057 (2)	0.8317 (2)	0.65084 (14)	0.0497 (5)	
C21	0.4573 (2)	0.9998 (2)	0.64844 (14)	0.0492 (5)	
C24	0.7062 (2)	1.0913 (2)	0.74752 (14)	0.0455 (4)	
C25	0.6252 (2)	1.0381 (2)	0.63910 (14)	0.0505 (5)	
C26	0.8751 (2)	1.1172 (2)	0.78925 (14)	0.0460 (4)	
C27	0.9750 (2)	1.1281 (2)	0.72391 (16)	0.0529 (5)	
C28	1.1338 (2)	1.1536 (3)	0.76154 (19)	0.0634 (6)	
C29	1.1953 (3)	1.1658 (3)	0.8629 (2)	0.0721 (7)	
C30	1.0978 (3)	1.1550 (3)	0.92885 (19)	0.0741 (7)	
C31	0.9385 (3)	1.1308 (3)	0.89236 (16)	0.0611 (5)	
H2	−0.1443	0.6829	0.8010	0.089*	
H3	−0.3548	0.6208	0.8691	0.103*	
H4	−0.5762	0.4584	0.7768	0.107*	
H5	−0.5886	0.3635	0.6131	0.113*	
H6	−0.3752	0.4230	0.5433	0.092*	
H7	−0.1244	0.5420	0.5498	0.066*	
H8	−0.0283	0.8109	0.6888	0.062*	
H13	0.001 (3)	0.421 (2)	0.677 (2)	0.090 (8)*	
H14	−0.1016	0.8399	0.5048	0.108*	
H16	0.4058	0.8964	0.8559	0.072*	
H17	0.4016	0.7947	1.0042	0.094*	
H18	0.2296	0.5714	1.0018	0.106*	
H19	0.0603	0.4538	0.8546	0.088*	
H20a	0.4042	0.7770	0.5861	0.060*	
H20b	0.4817	0.7994	0.7018	0.060*	
H21	0.3879	1.0336	0.5932	0.059*	
H25a	0.6590	0.9496	0.6126	0.061*	
H25b	0.6403	1.1174	0.5971	0.061*	
H27	0.9342	1.1181	0.6546	0.064*	
H28	1.1996	1.1626	0.7175	0.076*	
H29	1.3025	1.1813	0.8877	0.086*	
H30	1.1397	1.1641	0.9980	0.089*	
H31	0.8735	1.1235	0.9370	0.073*	

### Atomic displacement parameters (Å^2^)

**Table:**

	*U*^11^	*U*^22^	*U*^33^	*U*^12^	*U*^13^	*U*^23^
O14	0.0543 (9)	0.0779 (11)	0.0738 (11)	0.0015 (7)	−0.0006 (7)	0.0224 (8)
O15	0.0587 (10)	0.1559 (19)	0.0490 (9)	−0.0199 (10)	0.0048 (7)	0.0315 (10)
O22	0.0372 (7)	0.0582 (8)	0.0719 (9)	0.0069 (6)	0.0176 (6)	−0.0036 (6)
N10	0.0436 (8)	0.0540 (9)	0.0424 (8)	−0.0008 (7)	0.0114 (6)	0.0051 (6)
N13	0.0544 (10)	0.0427 (9)	0.0903 (13)	0.0053 (7)	0.0271 (9)	−0.0003 (9)
N23	0.0422 (9)	0.0560 (9)	0.0592 (10)	0.0041 (7)	0.0155 (7)	−0.0031 (7)
C1	0.0456 (10)	0.0480 (10)	0.0690 (13)	0.0034 (8)	0.0147 (9)	0.0102 (9)
C2	0.0495 (12)	0.1070 (19)	0.0675 (14)	0.0148 (12)	0.0178 (10)	0.0111 (13)
C3	0.0662 (16)	0.125 (2)	0.0872 (18)	0.0373 (16)	0.0352 (14)	0.0422 (17)
C4	0.0687 (17)	0.0772 (17)	0.146 (3)	0.0275 (14)	0.0540 (18)	0.0518 (18)
C5	0.0519 (14)	0.0548 (14)	0.171 (3)	−0.0052 (11)	0.0279 (17)	0.0121 (17)
C6	0.0554 (13)	0.0520 (12)	0.113 (2)	−0.0036 (10)	0.0177 (13)	−0.0087 (12)
C7	0.0489 (11)	0.0520 (11)	0.0580 (11)	−0.0035 (8)	0.0162 (9)	−0.0055 (9)
C8	0.0445 (10)	0.0541 (11)	0.0518 (11)	−0.0007 (8)	0.0082 (8)	0.0049 (8)
C9	0.0467 (11)	0.0674 (12)	0.0446 (10)	−0.0082 (9)	0.0066 (8)	0.0070 (9)
C11	0.0475 (10)	0.0532 (10)	0.0479 (10)	0.0138 (8)	0.0178 (8)	0.0106 (8)
C12	0.0527 (11)	0.0506 (11)	0.0713 (13)	0.0198 (9)	0.0284 (10)	0.0173 (9)
C16	0.0611 (12)	0.0728 (13)	0.0478 (11)	0.0184 (10)	0.0133 (9)	0.0085 (9)
C17	0.0833 (17)	0.113 (2)	0.0503 (13)	0.0401 (16)	0.0175 (11)	0.0187 (13)
C18	0.103 (2)	0.115 (2)	0.0788 (18)	0.0539 (19)	0.0466 (17)	0.0552 (17)
C19	0.0775 (16)	0.0741 (15)	0.0921 (19)	0.0343 (13)	0.0428 (14)	0.0422 (14)
C20	0.0439 (10)	0.0563 (11)	0.0471 (10)	0.0031 (8)	0.0152 (8)	0.0016 (8)
C21	0.0375 (9)	0.0577 (11)	0.0511 (10)	0.0055 (8)	0.0104 (8)	0.0083 (8)
C24	0.0401 (9)	0.0447 (9)	0.0519 (10)	0.0050 (7)	0.0147 (8)	0.0056 (7)
C25	0.0389 (10)	0.0609 (11)	0.0508 (10)	0.0030 (8)	0.0136 (8)	0.0092 (8)
C26	0.0398 (9)	0.0439 (9)	0.0531 (10)	0.0051 (7)	0.0110 (8)	0.0069 (8)
C27	0.0437 (10)	0.0561 (11)	0.0585 (11)	0.0078 (8)	0.0141 (9)	0.0046 (9)
C28	0.0406 (10)	0.0675 (13)	0.0827 (15)	0.0112 (9)	0.0182 (10)	0.0046 (11)
C29	0.0412 (11)	0.0707 (14)	0.0983 (19)	0.0137 (10)	0.0022 (11)	0.0102 (13)
C30	0.0646 (14)	0.0812 (16)	0.0663 (14)	0.0123 (12)	−0.0075 (11)	0.0163 (12)
C31	0.0546 (12)	0.0712 (13)	0.0562 (12)	0.0088 (10)	0.0104 (9)	0.0155 (10)

### Geometric parameters (Å, °)

**Table:**

O14—C8	1.412 (3)	C11—C16	1.386 (3)
O14—H14	0.8200	C12—C19	1.395 (3)
O15—C9	1.227 (3)	C16—C17	1.379 (4)
O22—N23	1.415 (2)	C16—H16	0.9300
O22—C21	1.446 (2)	C17—C18	1.383 (5)
N10—C9	1.345 (2)	C17—H17	0.9300
N10—C11	1.432 (2)	C18—C19	1.366 (4)
N10—C20	1.473 (2)	C18—H18	0.9300
N13—C7	1.475 (3)	C19—H19	0.9300
N13—C12	1.411 (3)	C20—C21	1.516 (3)
N13—H13	0.931 (19)	C20—H20a	0.9700
N23—C24	1.280 (3)	C20—H20b	0.9700
C1—C2	1.390 (3)	C21—C25	1.523 (3)
C1—C6	1.376 (3)	C21—H21	0.9800
C1—C7	1.516 (3)	C24—C25	1.497 (3)
C2—C3	1.377 (4)	C24—C26	1.470 (3)
C2—H2	0.9300	C25—H25a	0.9700
C3—C4	1.363 (4)	C25—H25b	0.9700
C3—H3	0.9300	C26—C27	1.395 (3)
C4—C5	1.363 (5)	C26—C31	1.388 (3)
C4—H4	0.9300	C27—C28	1.378 (3)
C5—C6	1.404 (4)	C27—H27	0.9300
C5—H5	0.9300	C28—C29	1.364 (4)
C6—H6	0.9300	C28—H28	0.9300
C7—C8	1.529 (3)	C29—C30	1.385 (4)
C7—H7	0.9800	C29—H29	0.9300
C8—C9	1.522 (3)	C30—C31	1.381 (4)
C8—H8	0.9800	C30—H30	0.9300
C11—C12	1.396 (3)	C31—H31	0.9300
			
C8—O14—H14	109.00	C17—C16—H16	120.00
N23—O22—C21	108.51 (14)	C16—C17—C18	119.5 (2)
C9—N10—C11	122.14 (16)	C16—C17—H17	120.00
C9—N10—C20	117.82 (15)	C18—C17—H17	120.00
C11—N10—C20	119.62 (15)	C17—C18—C19	120.5 (3)
C7—N13—C12	117.66 (16)	C17—C18—H18	120.00
C7—N13—H13	109.8 (17)	C19—C18—H18	120.00
C12—N13—H13	110.1 (16)	C12—C19—C18	121.2 (3)
O22—N23—C24	108.88 (16)	C12—C19—H19	119.00
C2—C1—C6	117.1 (2)	C18—C19—H19	119.00
C2—C1—C7	122.4 (2)	N10—C20—C21	114.08 (15)
C6—C1—C7	120.5 (2)	N10—C20—H20a	109.00
C1—C2—C3	121.6 (2)	N10—C20—H20b	109.00
C1—C2—H2	119.00	C21—C20—H20a	109.00
C3—C2—H2	119.00	C21—C20—H20b	109.00
C2—C3—C4	120.8 (3)	H20a—C20—H20b	108.00
C2—C3—H3	120.00	O22—C21—C20	109.67 (15)
C4—C3—H3	120.00	O22—C21—C25	103.98 (14)
C3—C4—C5	119.1 (3)	O22—C21—H21	110.00
C3—C4—H4	120.00	C20—C21—C25	111.47 (15)
C5—C4—H4	120.00	C20—C21—H21	111.00
C4—C5—C6	120.5 (3)	C25—C21—H21	111.00
C4—C5—H5	120.00	N23—C24—C25	113.70 (17)
C6—C5—H5	120.00	N23—C24—C26	120.91 (17)
C1—C6—C5	120.9 (3)	C25—C24—C26	125.39 (16)
C1—C6—H6	120.00	C21—C25—C24	100.10 (15)
C5—C6—H6	120.00	C21—C25—H25a	112.00
N13—C7—C1	113.45 (17)	C21—C25—H25b	112.00
N13—C7—C8	109.22 (16)	C24—C25—H25a	112.00
N13—C7—H7	107.00	C24—C25—H25b	112.00
C1—C7—C8	112.31 (15)	H25a—C25—H25b	109.00
C1—C7—H7	107.00	C24—C26—C27	119.42 (17)
C8—C7—H7	107.00	C24—C26—C31	121.77 (19)
O14—C8—C7	108.40 (16)	C27—C26—C31	118.82 (19)
O14—C8—C9	109.81 (16)	C26—C27—C28	120.3 (2)
O14—C8—H8	109.00	C26—C27—H27	120.00
C7—C8—C9	112.00 (15)	C28—C27—H27	120.00
C7—C8—H8	109.00	C27—C28—C29	120.5 (2)
C9—C8—H8	109.00	C27—C28—H28	120.00
O15—C9—N10	122.11 (18)	C29—C28—H28	120.00
O15—C9—C8	119.30 (18)	C28—C29—C30	119.9 (2)
N10—C9—C8	118.48 (16)	C28—C29—H29	120.00
N10—C11—C12	119.65 (17)	C30—C29—H29	120.00
N10—C11—C16	119.64 (18)	C29—C30—C31	120.3 (2)
C12—C11—C16	120.59 (19)	C29—C30—H30	120.00
N13—C12—C11	120.15 (19)	C31—C30—H30	120.00
N13—C12—C19	121.85 (19)	C26—C31—C30	120.2 (2)
C11—C12—C19	117.9 (2)	C26—C31—H31	120.00
C11—C16—C17	120.3 (2)	C30—C31—H31	120.00
C11—C16—H16	120.00		
			
O14—C8—C9—O15	−15.0 (3)	C7—C8—C9—O15	105.5 (2)
O14—C8—C9—N10	161.30 (17)	C7—C8—C9—N10	−78.2 (2)
O22—N23—C24—C25	0.4 (2)	C9—N10—C11—C12	42.8 (3)
O22—N23—C24—C26	179.36 (16)	C9—N10—C11—C16	−141.1 (2)
O22—C21—C25—C24	20.06 (17)	C9—N10—C20—C21	74.2 (2)
N10—C11—C12—N13	−1.1 (3)	C11—N10—C9—O15	−172.6 (2)
N10—C11—C12—C19	175.89 (19)	C11—N10—C9—C8	11.3 (3)
N10—C11—C16—C17	−174.9 (2)	C11—N10—C20—C21	−113.19 (18)
N10—C20—C21—O22	58.2 (2)	C11—C12—C19—C18	−1.2 (4)
N10—C20—C21—C25	172.78 (15)	C11—C16—C17—C18	−0.7 (4)
N13—C7—C8—O14	162.39 (16)	C12—N13—C7—C1	−79.9 (2)
N13—C7—C8—C9	41.1 (2)	C12—N13—C7—C8	46.2 (2)
N13—C12—C19—C18	175.7 (3)	C12—C11—C16—C17	1.1 (4)
N23—O22—C21—C20	97.71 (17)	C16—C11—C12—N13	−177.1 (2)
N23—O22—C21—C25	−21.61 (18)	C16—C11—C12—C19	−0.1 (3)
N23—C24—C25—C21	−13.3 (2)	C16—C17—C18—C19	−0.7 (5)
N23—C24—C26—C27	−163.20 (18)	C17—C18—C19—C12	1.7 (5)
N23—C24—C26—C31	17.0 (3)	C20—N10—C9—O15	−0.1 (3)
C1—C2—C3—C4	−0.8 (5)	C20—N10—C9—C8	−176.28 (15)
C1—C7—C8—O14	−70.8 (2)	C20—N10—C11—C12	−129.51 (19)
C1—C7—C8—C9	167.85 (17)	C20—N10—C11—C16	46.5 (3)
C2—C1—C6—C5	−1.8 (4)	C20—C21—C25—C24	−98.02 (17)
C2—C1—C7—N13	67.0 (3)	C21—O22—N23—C24	13.9 (2)
C2—C1—C7—C8	−57.4 (3)	C24—C26—C27—C28	179.47 (19)
C2—C3—C4—C5	−1.1 (5)	C24—C26—C31—C30	180.0 (2)
C3—C4—C5—C6	1.5 (4)	C25—C24—C26—C27	15.6 (3)
C4—C5—C6—C1	0.0 (4)	C25—C24—C26—C31	−164.2 (2)
C6—C1—C2—C3	2.2 (4)	C26—C24—C25—C21	167.78 (17)
C6—C1—C7—N13	−111.5 (2)	C26—C27—C28—C29	1.2 (4)
C6—C1—C7—C8	124.0 (2)	C27—C26—C31—C30	0.1 (3)
C7—N13—C12—C11	−73.2 (2)	C27—C28—C29—C30	−1.1 (4)
C7—N13—C12—C19	110.0 (2)	C28—C29—C30—C31	0.5 (4)
C7—C1—C2—C3	−176.4 (2)	C29—C30—C31—C26	0.0 (4)
C7—C1—C6—C5	176.8 (2)	C31—C26—C27—C28	−0.7 (3)

### Hydrogen-bond geometry (Å, °)

**Table:**

Cg4 is the centroid of the C26–C31 ring.

**Table:**

*D*—H···*A*	*D*—H	H···*A*	*D*···*A*	*D*—H···*A*
O14—H14···O15	0.82	2.14	2.632 (3)	118
C16—H16···O22	0.93	2.44	3.206 (3)	140
C25—H25a···O14^i^	0.97	2.59	3.493 (3)	154
C27—H27···O15^ii^	0.93	2.40	3.302 (3)	162
C28—H28···O22^i^	0.93	2.52	3.152 (2)	125
C19—H19···Cg4^iii^	0.93	2.78	3.534 (3)	139
N13—H13···Cg4^iii^	0.93 (2)	2.77 (2)	3.671 (3)	164

Symmetry codes: (i) *x*+1, *y*, *z*; (ii) −*x*+1, −*y*+2, −*z*+1; (iii) *x*−1, *y*−1, *z*.
